# Biomarker development perspective: Exploring comorbid chronic pain in depression through deep transcranial magnetic stimulation

**DOI:** 10.2478/jtim-2023-0145

**Published:** 2024-05-21

**Authors:** Peijun Ju, Di Zhao, Le Ma, Jinghong Chen

**Affiliations:** Shanghai Mental Health Center, Shanghai Jiao Tong University School of Medicine, Shanghai, China; Shanghai Institute of Traditional Chinese Medicine for Mental Health, Shanghai, China; Shanghai Key Laboratory of Psychotic Disorders, Shanghai, China

## Introduction

Pain is a complex and multifaceted experience that involves both sensory and emotional components. Globally, over 20% of adults suffer from diverse forms of chronic pain, which commonly coexist with comorbidities such as depression as their negative long-term health outcomes, leading to a reduction in their overall quality of life.^[1,2]^ It is generally observed that when depression and pain coexist, they tend to be more resistant to pharmacological treatment.^[3]^ Identifying effective treatments of chronic pain and depression in comorbidity is of paramount priority. To be specific, improving the identification, evaluation, and management of comorbid pain can potentially enhance the effectiveness of regulating negative emotions, and vice versa. Also, it is imperative to identify biological markers to characterize the different status in comorbid condition of pain and depression by comparing the specificity of responses in treatment processes.

Actually, there are no established clinical biomarkers for assessing treatment response in the majority of brain stimulation techniques. Clinical response to brain stimulation treatments for depression is also highly variable. Some neural biomarkers have been developed *via* transcranial magnetic stimulation (TMS) in concurrent combination with imaging-based localization (fMRI), electroencephalography (EEG) or measurement in intracortical and corticospinal electrophysiology for treatment response in depression.^[4,5]^ Utilizing such measures could enable the evaluation of the extent to which the treatment effectively engages the intended targets, and they could also serve as early indicators of treatment outcome. However, it is important to note that a significant number of individuals with depression also experience chronic pain symptoms, which have been linked to less favorable treatment outcomes with repetitive transcranial magnetic stimulation (rTMS). This highlights the necessity for a deeper comprehension of the shared pathophysiology between chronic pain and depression, as well as the development of innovative treatment approaches that target both conditions simultaneously. In addition, the identification of neurophysiological biomarkers associated with chronic pain have also been greatly attributed to the development of an rTMS protocol specifically designed to target.^[6,7]^ Therefore, we suggest that additional research is required to identify and understand the interplay between distinct and shared biomarkers implicated in the comorbidity of depression and pain. Considering that individuals with treatment-resistant depression and chronic pain often rely on analgesics for pain management, the introduction of a novel TMS therapeutic approach may potentially reduce the dependence on medication for these complex comorbid patients.

## The remarkable therapeutic efficacy of dtms in treating depression

Deep transcranial magnetic stimulation (dTMS) has recently been proposed as an alternative non-invasive procedure and has been demonstrated to target deeper and broader brain regions involved in neuropsychiatric diseases. It was proven effective and well-tolerated for depression in a large scale, double-blinded, multicenter randomized controlled trial.^[8]^ Further treatment using dTMS during the continuation phase of depression was shown to be clinically effective, even for acute phase non-responders. In real clinical practice settings, the response and remission rates for treatment-resistant depressive patients have been even more substantial. Greater than 4 in 5 achieve response and approximately 2 in 3 achieve full remission by dTMS treatment. Furthermore, more than half of patients maintain response at 90 days, following the start of the treatment.^[9]^ A randomized controlled trial (RCT) comparing H1-coil and Figure-8 TMS demonstrated superior outcomes for dTMS (Response: OR = 2.33; 95% CI: 1.04–5.21, *P* = 0.040). The reduction in Hamilton Depression Rating Scale 17(HAM-D17) scores was notable, with a 59% decrease in the H1-coil group, a 41% decrease in the 8-coil group (*P* = 0.048), and a 17% decrease in the control group (*P* < 0.001 *vs*. H1-coil; *P* = 0.003 *vs*. 8-coil).^[10]^ Recently, a meta-analysis demonstrated that the reduction in depression severity had a significantly (dTMS: 1.55 (1.17–1.94); k = 8; *n* = 183, 0.97 (0.70–1.25); k = 12; *n* = 168, *P* = 0.002) larger effect size after 10 sessions of dTMS with H1-coil (pooled g = 1.55) relative to rTMS with F8-coil.^[11]^ These results collectively indicated that dTMS has demonstrated more pronounced efficacy compared to traditional TMS in treating treatment-resistant depression that does not respond to medication.

Despite dTMS demonstrating remarkable advantages in the treatment of treatment-resistant depression, there is currently no research on the efficacy of dTMS for comorbidities of depression and pain. Until recently, a pilot study on the treatment of comorbid anxiety and pain has provided preliminary evidence that dTMS has the potential to treat comorbidities of pain and negative emotions. Ju *et al*. focused on assessing the efficacy of dTMS in treating the comorbidity of chronic pain with anxiety.^[12]^ The active dTMS treatment showed significant add-on analgesic effects compared to the waiting-list control group. Moreover, the state-trait anxiety score showed a moderate correlation with pain relief. The results preliminarily suggested that the alleviation of laser-induced pain perception through dTMS therapy which aimed at the medial prefrontal cortex (mPFC) and anterior cingulate cortex (ACC) regions might have been linked to the concurrent regulation of anxiety in those regions, as evidenced by the modest correlation between the two factors. It is well known that the brain supports the above complex cyclical interaction between pain and specific emotional states. For example, brain regions involved in pain processing, such as the anterior cingulate cortex, insula, and amygdala, are also implicated in emotional regulation and the experience of emotions.^[13,14,15]^ There is a suggestion that there might be shared neural circuitry between chronic pain and depression, and certain neurophysiological characteristics within tshis circuitry could define a specific subtype of depression that is more prone to developing comorbid pain and more responsive to dTMS treatment. Subsequent studies should systematically investigate potential common targets for dTMS treatment of depression, such as the primary motor cortex, ventromedial prefrontal cortex, or the anterior cingulate cortex, all of which have been associated with pain processing or pain relief following dTMS. Therefore, a multi-target dTMS approach for the simultaneous treatment of depression and chronic pain holds promise as a viable alternative therapeutic strategy.

## Biomarker discovery in comorbid depression and pain through dtms treatment

Considering the wide range of potential anatomical and functional targets of dTMS, how can we effectively assess its clinical utility and potential therapeutic effects? Identifying a reliable neural measure suitable for modification through brain stimulation poses the initial and potentially most challenging obstacle. Numerous studies employing a pre-post-trial design have reported neural or biological measures that are correlated with treatment response. However, conventional verbal report scales like the HAM-D may not adequately capture significant clinical changes in domains such as negative self-talk, optimism, and self-confidence.^[16]^ Consequently, it is evident that at the symptom level, two patients meeting diagnostic criteria for depression may not share a single common criterion. Depression lacks a consistent neural phenotype, rendering the task of optimizing brain stimulation for depression as a whole potentially unachievable. Moreover, these standard scales fail to differentiate distinct components of depression, such as anhedonia or emotional dysregulation, despite their distinct neural underpinnings. Thus, preliminary research is necessary to identify clusters of patients with stable phenotypes. Herein, a group of depressed patients who exhibit prominent comorbid pain features could be focused. Due to the synchronous improvement of the anatomical foundation and symptom treatment in the comorbidity of depression and pain, pain relief can serve as an easily assessable stable phenotype for identifying changing markers before and after treatment, thus possessing diagnostic and therapeutic significance. With more complex approaches like univariate fMRI, the stability of measurement becomes imperative. The within-subject reliability of certain neural activation measurements in relationship with pain can significantly vary based on imaging techniques, the measured brain region, and analysis methods in comorbidity patients.^[17]^

In the realm of depression, the utilization of rTMS offers promising prospects for personalized treatment strategies, notably by leveraging neurophysiological and neuroimaging biomarkers. Following rTMS intervention, notable enhancements in the activity of the dorsolateral prefrontal cortex (DLPFC), hippocampus (HPC), and orbitofrontal cortex (OFC), along with the boost in HPC activity indicated by delta changes, were closely linked to the amelioration of depressive outcome variables.^[18]^ Neurophyiological measures by TMS-EEG also emerges as potential disease specific biomarkers aiding in predicting treatment response and monitoring post-treatment changes.^[5]^ For instance, excitability indices in TMS-EEG, such as N100 and N45, serve as biomarkers for the diagnosis and treatment of depression. Further research is warranted to authenticate the imaging and electrophysiological biomarkers linked to dTMS in the treatment of comorbid depression and pain. Dysregulations in various neurotransmitters are commonly investigated for a comprehensive understanding biomarkers of chronic pain. For example, glutamate could be assessed by active motor threshold (AMT), resting motor threshold (RMT), motor evoked potential (MEP), intracortical facilitation (ICF), GABA were evaluated by MEP, short-interval intracortical inhibition (SICI), cortical silent period (CSP), short-latency afferent inhibition (SAI), Long-latency afferent inhibition (LAI). Acetylcholine was measured by SAI. Voltage-gated sodium channels were examined by AMT and RMT. Serotonin were gauged by MEP, and norepinephrine were assessed by MEP.^[19,20,21]^ Patients with chronic primary pain tend to exhibit higher motor thresholds and lower MEP amplitude, SICI, and ICF compared to healthy controls.^[22]^ Post-interventions, both SICI and ICF tend to normalize. Although no direct correlations were observed between TMS outcomes and quantitative pain measures, positive associations were found between pain catastrophizing and SICI and ICF, depression and SICI, and ICF, and illness impact on functioning and quality of life.^[23]^ These findings suggest anomalies in intracortical GABAergic inhibition and glutamatergic facilitation, which are linked to aspects of suffering and function. Hence, intracranial methods, such as animal models and human intracranial EEG, can also offer a more reliable assessment to enhance the evaluation of biomarker validity. Furthermore, simultaneous scalp and intracranial EEG may allow for direct comparisons between noninvasive biomarkers and their neural correlates within the context of chronic pain neuropathophysiology, with special reference to the “pain matrix”. Pain elicits emotional responses in individuals.^[24]^ The intensity and nature of these emotional responses can vary depending on factors such as the type and severity of pain. One proposed solution to address this issue involves evaluating the impact of dTMS stimulation interventions on behavioral or symptom measures that are known to be associated with specific neural circuits. Instead of assessing the overall effects of an intervention on major depressive disorder, clinical trials can focus on the specific neural circuits targeted by the intervention and measure changes in pain-related symptoms. By establishing a reliable and consistent neural phenotype or dimension, subsequent studies can explore two critical factors: the relationship between pain relief and treatment response for depression, and the potential to modify the pain relief phenotype using specific brain stimulation interventions. In the latter case, data-driven approaches can identify dimensions that go beyond traditional treatment categories, facilitating the development of experimental medicine studies that specifically target these dimensions.

In addition to imaging and electrophysiological measures, neuroinflammatory factors can potentially serve as biomarkers for assessing the response to dTMS treatment in individuals with comorbid depression and pain. Mental illnesses are closely linked to the inflammatory response and neurotransmitter activities within the body, which not only manifest in the peripheral system but also impact the central neural circuits that are the primary focus of neuromodulation therapy.^[25]^ Various neuromodulation techniques have been found to influence the body’s inflammatory response and neurotransmitter levels during their application.^[26]^ Therefore, it is reasonable to hypothesize that monitoring peripheral inflammatory factors and neurotransmitters could serve as potential biomarkers, influenced by factors such as stimulation frequency, stimulation pulse width, stimulation duration, stimulation intensity, and duration in regular TMS treatments. In this way, it is speculated that comprehensive data should also be available regarding the molecular changes induced by dTMS treatment, as well as predictive molecular biomarkers of dTMS response could be identified. In order to optimize the effectiveness of dTMS treatment, it is crucial to accumulate substantial clinical evidence and apply data science techniques, with a specific emphasis on long-term data collection including the utilization of neuroinflammatory biomarkers. The utilization of such information has the potential to enhance our understanding of the biological mechanisms underlying dTMS treatment. It is known that brain-derived neurotrophic factor (BDNF) serves as a neurobiological marker indicating both schizophrenia and depression and plays a role in assessing the effectiveness of rTMS treatments for these mental disorders.^[27]^ And so forth, molecules such as GABA and BDNF in depression, β-endorphin in chronic pain have shown promise as molecular markers of dTMS. Additionally, the reduction of pro-inflammatory factors and microglial function may contribute to discovery of biomarkers for dTMS Treatment Response in the comorbidity of depression and pain.

## Addressing challenges in clinical practice: concetual suggestions, pratical methods and future directions for dTMS

Overall, response rates (≥50% symptom improvement) in dTMS typically range between 15% and 50%, indicating substantial potential for enhancement in the treatment of comorbid depression and pain.^[11]^ While the coil configuration in dTMS remains relatively fixed, requiring minimal adjustments in position and orientation, the administration of dTMS presents an extensive range of customizable parameters, including stimulation intensity, frequency, pulse frequency, number of pulses, sessions, and intersession intervals. Drawing from conventional TMS expertise, a personalized protocol for dTMS can be applied, utilizing established methods from personalized conventional TMS treatment parameters. The effectiveness of personalized protocols, derived from neuroimaging or electroencephalography techniques, has been suggested to surpass that of standard TMS approaches as mentioned earlier.^[28]^ Likewise, concurrent dTMS-EEG or dTMS-fMRI approaches may contribute to refining subject-specific stimulation intensities. Stimulation frequencies can be tailored to individual firing frequencies.^[29]^ Individualized dTMS treatment takes into account diverse rhythmic firing patterns, suggesting that the stimulation frequency for clinical effects is subject-specific and customizable. For dTMS-EEG, optimizing efficacy involves tuning the stimulation to instantaneous phase or power values reflecting heightened excitability states. Additionally, stimulation intensity, the sole parameter derived from subject-specific characteristics, is determined by gradually increasing intensity until hand muscle contraction, known as the motor threshold.

Neuroimaging has unveiled that TMS effects extend beyond the immediate site, influencing distributed brain networks. Multimodal imaging enables the identification of patient-specific stimulation parameters, highlighting the significant inter-individual variation in brain network architecture, particularly in the prefrontal cortex, where unique connectome fingerprints may influence treatment outcomes of TMS across various disorders.^[30]^ Hence, large, potentially multicenter, prospective clinical trials in which patients with highly treatment-resistant depression are randomized to receive standard TMS procedures.^[31]^ Similarly, dTMS can be combined with fMRI for neuronavigation in practical applications, and the design of RCTs can be employed to explore treatment outcomes.

DTMS treatment for comorbid depression and pain is currently in its exploratory phase, bringing forth several crucial considerations:(1) It is essential to replicate findings across diverse patient groups experiencing depression, pain, and comorbid depression and pain, considering various clinical phenotypes. This is crucial to establish the reliability of dTMS biomarkers for comorbid depression and pain. While some studies suggest the effectiveness of dTMS in depression, the outcomes for comorbid depression and pain remain variable and unpredictable. Further investigations are imperative to ensure the consistency of efficacy and identify optimal candidates for this treatment, particularly in the context of comorbid depression and pain. Simultaneously, employing pharmacological methods is essential to gain deeper insights into the neurobiological targets of the dTMS indexes. (2) Safety and Potential Adverse Effects The persistent safety profile and potential long-term side effects of dTMS are not fully understood. Despite short-term studies suggesting a level of safety, evaluating potential risks and adverse effects linked to extended use is crucial. (3) Identification of Optimal Stimulation Parameters: In the case of dTMS, the process entails the selection of the stimulation frequency, intensity, and other related parameters. The most efficient stimulation parameters remain uncertain, prompting further investigation to determine ideal, uniform, and replicable parameters for clinical applications. (4) The exact mechanism through which dTMS influences depression and pain in comorbidity is not completely clarified. A more profound comprehension of its mode of operation has the potential to improve the optimization of clinical applications. (5) Personalizing Therapeutic Approaches for Comorbid Depression and Pain: Recognizing the unique circumstances of each patient, tailored therapeutic strategies are essential. Leveraging advancements in brain imaging and biomarkers can guide treatment decisions, enhancing overall effectiveness. Overcoming these challenges will facilitate the integration of dTMS into clinical practice, providing more effective solutions for comorbid depression and pain.

## Potential side effects of dTMS treatment

Currently, no serious side effects necessitated dTMS treatment interruption, such as seizures, and the absence of reporting milder adverse events, like short-lived headaches after stimulation.^[32]^ In the context of depression treatment, most studies did not adequately report adverse effects, which encompassed headaches and discomfort from the stimulus. Similarly, for pain treatment, no major side effects were observed. However, it is noteworthy that during each treatment session, a recommended side effect assessment using a dedicated questionnaire was suggested to monitor any study-related complications. Patients were instructed to report potential neurological side effects, such as headaches, nausea, sleepiness, and local pain.^[33,34]^ The absence of information regarding the likelihood of serious adverse events constitutes crucial gaps that could enhance more informed decision-making and assist in managing unrealistic expectations. Therefore, future assessments of side effects should be further strengthened.

## Conclusion

Brain stimulation interventions by dTMS could be designed to target specific pain phenotypes in depression. Neural pathways and neurotransmitters involved in pain transmission and emotional processing intersect, influencing how pain and emotion are perceived and modulated. Therefore, targeting the overlapping neural mechanisms between pain and emotion is crucial in developing holistic approaches to pain management. Further studies are necessary to characterize the interaction of separate or common brain circuits that may be involved, and investigation should attempt to more clearly distinguish these elements of pain. Based on the background mentioned above, techniques such as dTMS that target the regulation of neural circuits offer the advantage of precise treatment in cases where pain and emotional comorbidity are present. The common effect brain regions and connections may become good targets for treatment. In comparison to other TMS techniques, dTMS offers several advantages. This device outperforms traditional TMS by achieving a better balance between field spread and penetration, resulting in deeper penetration and more accurate targeting localization. Considering the high occurrence of comorbid chronic pain and depression, and recognizing their shared underlying pathophysiology, it becomes crucial to identify innovative targets and integrated interventions utilizing dTMS for the treatment of both pain and depression concurrently, instead of separately addressing each symptom. It can target deeper brain structures, including the prefrontal cortex and limbic system, which are involved in emotional regulation, other than the surface of the brain. This makes it particularly useful for treating a range of neuropsychiatric disorders such as depression, anxiety,^[35]^ addiction,^[36]^ obsessive-compulsive disorder.^[37]^ In clinic, one of the benefits of dTMS is that it is generally well-tolerated by patients, with few reported side effects. ^[38]^ Overall, the future trajectory of dTMS research in depression comorbid with pain holds great promise.
